# Expression of L-type amino acid transporter 1 is a poor prognostic factor for Non-Hodgkin’s lymphoma

**DOI:** 10.1038/s41598-021-00811-8

**Published:** 2021-11-04

**Authors:** Narangerel Jigjidkhorloo, Kohsuke Kanekura, Jun Matsubayashi, Daigo Akahane, Koji Fujita, Keiki Oikawa, Atsushi Kurata, Masakatsu Takanashi, Hitoshi Endou, Toshitaka Nagao, Akihiko Gotoh, Oyundelger Norov, Masahiko Kuroda

**Affiliations:** 1 Department of Molecular Pathology, Tokyo Medical University, 6-1-1 Shinjuku, Shinjuku-ku, Tokyo, 160-8402 Japan; 2 Center of Hematology and Blood & Marrow Transplantation, The First Central Hospital of Mongolia, Ulaanbaatar, 14210 Mongolia; 3 Department of Anatomical Pathology, Tokyo Medical University Hospital, 6-7-1 Nishi-Shinjuku, Shinjuku-ku, Tokyo, 160-0023 Japan; 4 Department of Hematology, Tokyo Medical University Hospital, 6-7-1 Nishi-Shinjuku, Shinjuku-ku, Tokyo, 160-0023 Japan; 5 J-Pharma Co., Ltd., 75-1 Ono-cho, Tsurumi-ku, Yokohama, Kanagawa 230-0046 Japan

**Keywords:** Cancer, Drug discovery, Diseases, Oncology

## Abstract

L-type neutral amino acid transporter 1 (LAT1) is a heterodimeric membrane transport protein involved in neutral amino acid transport. LAT1 is highly expressed in various malignant solid tumors and plays an essential role in cell proliferation. However, its role in malignant lymphoma remains unknown. Here, we evaluated LAT1 expression level in tissues from 138 patients with Non-Hodgkin lymphoma (NHL). Overexpression of LAT1 was confirmed in all types of NHL and we found that there is a significant correlation between the level of LAT1 expression and lymphoma grade. The LAT1 expression was higher in aggressive types of lymphomas when compared with static types of lymphomas, suggesting that active tumor proliferation requires nutrient uptake via LAT1. The expression level of LAT1 was inversely correlated with patients’ survival span. Furthermore, pharmacological inhibition of LAT1 by a specific inhibitor JPH203 inhibits lymphoma cell growth. In conclusion, our study demonstrated that LAT1 expression can be used as a prognostic marker for patients with NHL and targeting LAT1 by JPH203 can be a novel therapeutic modality for NHL.

## Introduction

Non-Hodgkin lymphomas (NHLs) are a heterogeneous group of more than 100 of lymphoid neoplasms that differ in clinical features, dynamics of their course, response to treatment, and prognosis. NHLs affect 510,000 people/year and cause 249,000 death/year worldwide^1^. NHLs are classified into the indolent type (low-grade) and the aggressive type (intermediate and high-grade). The precise grading is the crucial task to arrange the most suitable and more specific treatment options for each patient. Some types of indolent lymphoma can be followed by observation only whereas an aggressive lymphoma requires immediate treatment by intensive multidrug chemotherapy alone or in combination with advanced treatment options such as molecular-targeted therapy and hematopoietic stem cell transplantation^2^. Therefore, the biomarkers whose expressions correlate with disease staging and help classification of NHLs have been sought and established. The diagnosis guideline of NHLs is annually updated by World Health Organization (WHO)^3^. Most established prognostic biomarker is Ki-67 proliferation index which is used to distinguish indolent and aggressive lymphoma^4,5^. Other biomarkers such as cell surface markers and amplification of oncogenic genes are used in combination to precisely diagnose the type of lymphoma. Although many diagnostic biomarkers have been identified and clinically used, there are still difficult and uncertain cases of NHL which need further characterization. Therefore, researchers have been keen on developing more accurate and reliable diagnostic markers of lymphoma.

The major hallmark of cancer cells is their spontaneous and unlimited proliferation. Rapid cellular proliferation requires a huge amount of nutrient supply including amino acids. Essential amino acids are uptaken by amino acid transporters on the cell membrane. The group of transporters that uptakes neutral amino acids in a sodium-independent manner is called L-type neutral amino acid transporters (LATs)^6^. To date, four LATs have been identified^7–10^. Among them, L-type neutral amino acid transporter 1 (LAT1) is responsible for the transport of large neutral amino acids such as leucine, isoleucine, valine, phenylalanine, tyrosine, tryptophan, methionine, and histidine^7^. Importantly, LAT1 was found to be overexpressed in a wide range of human malignant solid tumors such as lung, prostate, breast, and kidney cancers^11–14^. Its expression level was significantly associated with the tumor stage, poor prognosis and shorter survival. On the contrary, several studies reported that LAT1 was not detected by immunohistochemical staining in most normal tissues excluding activated lymphocytes^15^, uterine glands^16^, and the basal layer of the esophageal and uterine mucosa^17,18^. Therefore, tumor-specific LAT1 expression can be a good biomarker for malignant tumors and also LAT1 can be an excellent molecular target for chemotherapy. In the hematology field, there are no previous studies evaluating the expression level of LAT1 in lymphoma. In this study, we aimed to investigate retrospectively the correlation between LAT1 expression in NHL and clinical outcome and subtype of lymphomas, and found a significant correlation between the expression of LAT1 and lymphoma grade. The expression level of LAT1 showed a negative correlation with patients’ survival span. Combined with the data that LAT1 inhibitor suppresses lymphoma cell growth, LAT1 can be a biomarker for NHL and a novel therapeutic target for NHL.

## Materials and methods

### Specimens

A total of 138 (132 lymph node, 4 lung tissue, 2 parathyroid gland) biopsy samples were obtained from patients with NHL who were diagnosed and treated at the Tokyo Medical University Hospital (Tokyo, Japan) between 2006 and 2018 under informed consent. And a total of 14 reactive lymph node biopsy samples were obtained and used for the study who were diagnosed other than lymphoma at the hospital. The study was conducted in accord with the Declaration of Helsinki and the study protocol was approved by the Institutional Review Board (IRB) of Tokyo Medical University (ID1333). All study procedures were performed according to the appropriate requirement and regulations. All samples were classified according to the WHO Classification of hematopoietic and lymphoid tissues^3^. 115 cases were diagnosed as B cell lymphomas and subclassified as follows; 30 cases were Diffuse large B-cell lymphomas (DLBCL), 69 cases were Follicular lymphomas (FL), 6 cases were Mantle cell lymphoma (MCL), 5 cases were Marginal zone lymphoma (MZL), 3 cases were Burkitt lymphoma (BL), 2 cases were Small lymphocytic lymphoma (SLL). 23 cases were diagnosed as T and Natural killer cell lymphomas including 6 cases of Angioimmunoblastic lymphomas (AILT), 7 cases of Peripheral T-cell lymphoma (PTCL), 5 cases of Anaplastic large cell lymphoma (ALCL), and 5 cases of Extranodal Natural killer and T cell lymphoma nasal type (ENKTCL). As lymphoma grade, 59 cases were classified as aggressive grade and 73 cases as indolent grade, respectively. In addition, 6 cases with MCL were unclassified by grade.

The clinical data of 138 patients were reviewed by the following parameters: age and gender, the cancer stage, WHO histopathological classification of NHL, lymphoma grade (indolent and aggressive), International Prognostic Index (IPI) risk group expecting FL cases, Follicular Lymphoma International Prognostic Index 2 (FLIPI2) classification in FL cases, bone marrow and extranodal involvement, cytogenetic abnormality, serum lactate dehydrogenase (LDH) and hemoglobin level, the effect of treatment and outcome (survival/death).

### Immunohistochemistry

All specimens, fixed in formalin and embedded in paraffin, were cut into 4 µm thick sections. Deparaffinization and rehydration were performed through xylene and graded alcohol. Endogenous peroxidase activity was blocked by incubation with 0.3% hydrogen peroxide in methanol for 15 min. All sections were boiled in a pressure cooker in 10 mM Tris-1 mM EDTA pH 9.0 solution for 10 min at 121 °C and were cooled for an hour. After 3 times of rinsing in phosphate-buffered saline (PBS), the sections were incubated with primary antibodies, including mouse monoclonal anti-LAT1 (dilution of 1:3000; J-Pharma, Japan) or mouse monoclonal anti-Ki-67 (dilution of 1:50; MIB-1; Dako; Denmark) for 90 min at room temperature. For detection, REAL Envision Detection Systems (cat.no 5007; Dako; Denmark) was used for 30 min followed by chromagen staining using diaminobenzidine (DAB) for 3 min and finally counterstained by hematoxylin.

Immunofluorescent double staining of selected sections was carried out to identify co-expression of LAT1 and Ki-67. The staining process was conducted as described above, excluding the incubation with 0.3% hydrogen peroxide in methanol. The cocktails of mouse monoclonal anti-LAT1 antibody (dilution of 1:3000) and rabbit monoclonal anti-CD20 antibody (dilution of 1:100; EP4594; ab78237; Abcam; Japan), or rabbit monoclonal anti-CD3 antibody (dilution of 1:90; SP7; Nichirei Bioscience; Japan) or rabbit monoclonal anti-Ki67 antibody (dilution of 1:500; SP6; ab6667; Abcam; Japan) were used as primary antibody. The sections were incubated in mixture of Alexa Fluor 488 donkey anti-mouse IgG (H + L) (dilution of 1:500; A21201; Molecular Probes, Invitrogen; USA) and Alexa Fluor 594 donkey anti-rabbit IgG (H + L) (dilution of 1:500; A21207; Molecular Probes, Invitrogen; USA) for visualization. Also, 4′,6-diamidino-2-phenylindole (DAPI) (340-07971; Cellstain) was used for nuclear visualization. Normal lymph node tissue was used as a negative control. Images were captured and collected by Olympus FV-10i confocal microscopy (Olympus, Japan).

### Evaluation of immunohistochemistry

LAT1 expression was considered positive only if distinct cell membrane staining was present. First, the 3 most proliferative areas of the lymphoma were chosen at low magnification (×100) to minimize the problem of admixing of nonmalignant lymphocytes and macrophages. Further, the slides were examined at ×400 magnification to count LAT1 positive cells and negative cells on selected 3 areas, then the percentage of LAT1 positive cells was calculated and the average of three distinct areas was determined. The percentage of LAT1 positive cells were evaluated as follow: 0 = no positive immunostaining; 1 =  < 10%; 2 = 10–25%; 3 = 25–50% and 4 =  > 50% of tumor cell showing positive immunoreactivity. 1 and 2 scores were considered as LAT1 low expression, 3 and 4 scores were considered as LAT1 high expression. The Ki-67 percentage score was defined as the percentage of positively stained tumor cells (nuclear staining of any intensity) among the total malignant cells assessed in the areas used for evaluation of LAT1 positivity. At least 1000 cells were evaluated in each slide. The sections were assessed using a stereomicroscope from the Leica microsystems. Scoring calculations were performed in a blinded to each patient’s clinical status by the author. As reactive lymph node samples, LAT1 positive cells were evaluated as a percentage at each follicle and medulla or cortex area.

### Lymphoma cell growth assay

Human lymphoma cell lines, Raji cells, BJAB cells and U-937 cells were grown in RPMI-1640 medium (Gibco, Waltham, MA) supplemented with 10% fetal bovine serum (FBS) (Gibco) and 1% penicillin/streptomycin at 37 °C in 5% CO_2_. The cells were seeded (5000 cells/well) on a 96 well plate with serially diluted JPH-203, a specific inhibitor for LAT1 (Selleck Chemicals, Houston, TX), and incubated for 72 h. The cell viability was assessed by Cell-titer glo 2.0 (Promega, Fitchburg, WI) following the manufacturer’s instruction. HEK293 cells were maintained in Dulbecco’s modified eagle medium supplemented with 10% FBS and antibiotics. HEK293 cells were seeded (3000 cells/well) on a 96 well plate and treated with JPH-203 for 72 h, followed by viability analysis with Cell-titer glo 2.0.

### Western blot analysis

The cells were lysed in a lysis buffer (20 mM Tris–HCl pH7.4, 0.5% Nonidet-P40 and protease inhibitor cocktails), and applied to SDS-PAGE. The proteins were blotted onto Immobilon-P polyvinylidene fluoride (PVDF) membrane (Millipore, Burlington, MA). Immunoblotting was performed with anti-LAT1 antibody (J-Pharma) and anti-tubulin antibody (Santa Cruz Biotechnology, Dallas, TX).

### Statistical analysis

The relationship between different variables was analyzed using the non-parametric Spearman’s rank test. Overall survival (OS) rate was computed from the first day of diagnosis to the date of last follow-up or death by Kaplan–Meier method and survival differences were analyzed by the log-rank test. Survival data were analyzed by the Cox regression method. Factors significant in univariate analysis were examined by multivariable analysis. A cut-off index value differentiating two operating characteristics was determined by the receiver operating characteristics (ROC) curve. p-values less than 0.05 were considered to indicate a statistically significant difference. The SPSS 21.0 software was used to perform all statistical analyses (SPSS, Inc., USA).

### Consent for publication

We obtained the written forms of informed consent from all the patients regarding the use of their clinical data, following the ethical guideline of Tokyo Medical University. The data from patients were analyzed anonymously.

## Results

### Patient characteristics

The patient characteristics are summarized in Table 1. The 138 NHL patients included 72 men and 66 women, of the mean age 63.2 ± 13.7 years (range 28–91 years). 66.6% of the patient were diagnosed at the advanced stage of lymphoma (Clinical stage III, IV). According to the IPI scoring system, 92 patients were classified into the intermediate high and high risk group.

**Table 1:** Characteristics at diagnosis of patients with NHL.

	Patients (diagnosed in 2006–2018) n = 138	DLBCL n = 30	FL n = 69
**Age (years)**	28–91	36–90	31–91
Mean range	63.2 ± 13.7	64.8 ± 14.9	62.7 ± 13.1
**Sex**			
Male	72 (52.2%)	15 (50%)	31 (44.9%)
Female	66 (47.8%)	15 (50%)	38 (55.1%)
**Cancer stage**			
I	19 (13.8%)	4 (13.3%)	7 (10.1%)
II	27 (19.6%)	5 (16.7%)	15 (21.8%)
III	42 (30.4%)	7 (23.3%)	25 (36.2%)
IV	50 (36.2%)	14 (46.7%)	22 (31.9%)
**Serum LDH**			
Normal	78 (56.5%)	8 (26.7%)	52 (75.4%)
Elevated	60 (43.5%)	22 (73.3%)	17 (24.6%)
**Hemoglobin**			
Normal	103 (74.6%)	18 (60%)	56 (81.2%)
Decreased	35 (25.4%)	12 (40%)	13 (18.8%)
**IPI risk group**			
Low risk	6 (8.7%)	4 (13.3%)	
Low-intermediate risk	20 (29%)	2 (6.7%)	
High-intermediate risk	25 (36.2%)	11 (36.7%)	
High risk	18 (26.1%)	13 (43.3%)	
**FLIPI2 risk category**			
Low risk			16 (23.2%)
Intermediate risk			39 (56.2%)
High risk			14 (20.3%)
**Bone marrow involvement**			
Yes	29 (21%)	5 (16.7%)	12 (17.4%)
No	84 (60.9%)	19 (63.3%)	45 (65.2%)
Not done	25 (18.1%)	6 (20%)	12 (17.4%)
**Extranodal involvement**			
Yes	56 (40.6%)	15 (50%)	24 (34.8%)
No	82 (59.4%)	15 (50%)	45 (65.2%)
**Cytogenetic abnormality**			
Yes	63 (45.7%)	12 (40%)	44 (63.8%)
No	61 (44.2%)	17 (56.7%)	22 (31.9%)
Not done	14 (10.1%)	1 (3.3%)	3 (4.3%)
**LAT1 expression**			
Low	53 (38.4%)	1 (3.3%)	36 (52.2%)
High	85 (61.6%)	29 (96.7%)	33 (47.8%)
**Ki-67 expression**			
< 70%	120 (87%)	21 (70%)	68 (98.6%)
> 70%	18 (13%)	9 (30%)	1 (1.4%)

### Characterization of expression of LAT1 by NHL subtype and grade

**Figure 1:**
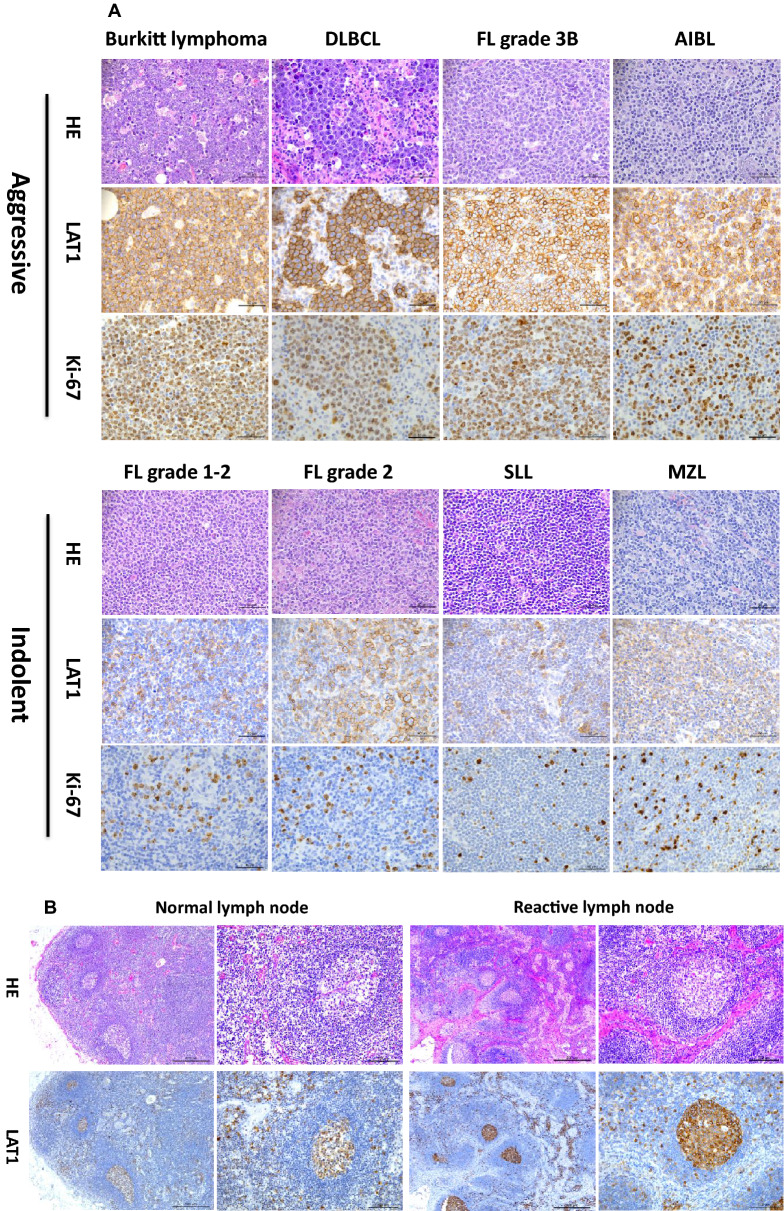
Representative images of Immunofluorescence double staining of lymphoma cells. (**A**) Representative images of HE staining, LAT1 and Ki-67 immunohistochemistry staining in selected cases of aggressive and indolent lymphomas. Aggressive lymphomas (Burkitt lymphoma, Diffuse large B-cell lymphoma (DLBCL), Follicular lymphoma (FL) grade 3B, Peripheral T-cell lymphoma (PTCL)) and indolent lymphomas (Follicular lymphoma (FL) grade 1–2 and grade 2, Small lymphocytic lymphoma (SLL), Marginal zone lymphoma (MZL)). Aggressive lymphomas on the upper row expressed higher levels of LAT1 than indolent lymphomas on the lower row. LAT1 expression levels and patterns were different on the classification of lymphoma aggressiveness. (**B**) LAT1 staining in the normal lymph node and reactive lymph node as negative controls. Normal lymphocytes did not express LAT1 excluding some activated lymphocytes in the germinal center of the follicle and medulla area. On the other hand, many lymphocytes, especially in the germinal center area, were strongly positive for LAT1 in reactive lymph node tissue. Scale bars indicate 50 µm (zoomed) or 500 µm.

**Figure 2:**
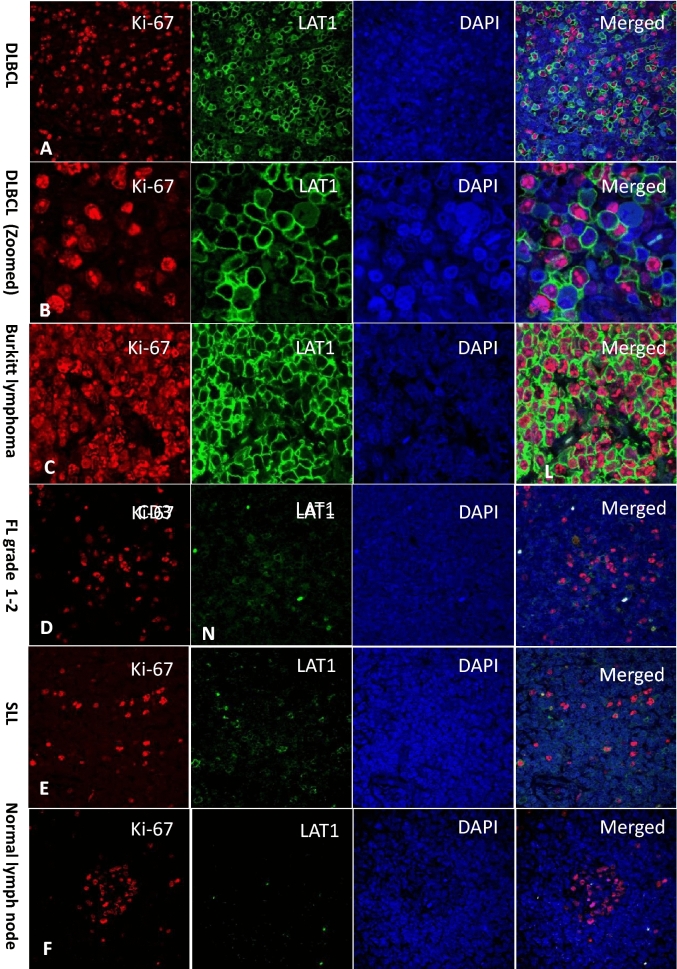
Representative images of Immunofluorescence double staining with LAT1 and Ki-67 of lymphoma cells. (**A**) Immunostaining of DLBCL tissue with anti-LAT1 antibody (red) or anti-Ki67 antibody (green). Nuclei were counter-stained by DAPI. (**B**) Zoomed images of the DLBCL tissue. (**C**) Immunostaining of Burkitt lymphoma. (**D**) Immunostaining of the follicular lymphoma cells. (**E**) Immunostaining of the SLL tissue. (**F**) Immunostaining of the normal lymph node tissue as a negative control. Scale bar, 50 µm.

**Figure 3:**
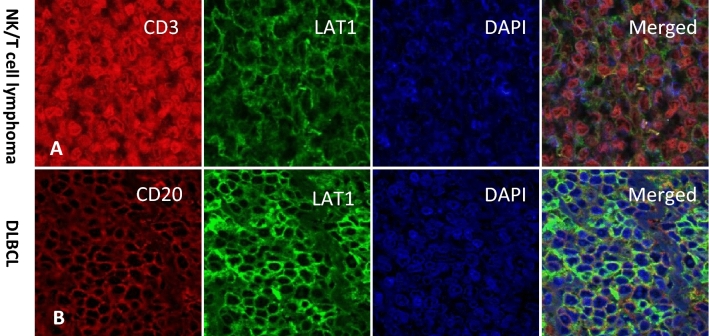
Representative images of immunofluorescence double staining of lymphoma cells with a T cell marker CD3 or a B cell marker CD20. (**A**) NK/T cell lymphoma cells co-expressed CD3 and LAT1. (**B**) DLBCL cells co-expressed CD20 and LAT1.

**Figure 4:**
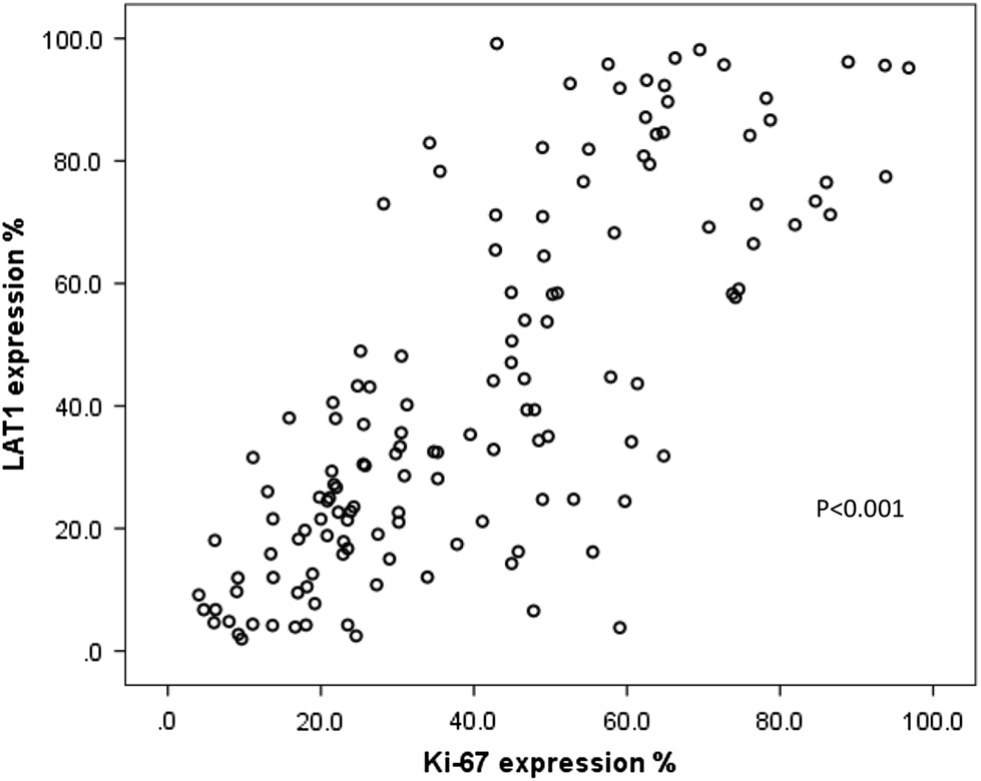
Correlation between LAT1 and Ki-67 expression. LAT1 expression level in NHL is strongly associated with Ki-67 expression which is cell proliferation marker (by the Spearman’s rank test r = 0.767, P < 0.001).

There were total 138 specimens with 10 subtypes of NHL. The number of 115 (83.5%) were B cell lymphoma cases and the number of 23 (16.5%) were T and NK cell lymphoma cases. All the specimens were evaluated by immunohistochemical analyses, and as expected, the LAT1 signal was detected at the plasma membrane. Representative pictures of the immunohistochemical staining of LAT1 and Ki-67 in aggressive and indolent lymphomas and normal lymph node (negative control) are shown in the Fig. 1A,B. Immunofluorescent double staining showed that actively proliferating Ki-67 positive tumor cells are positive for LAT1 (Fig. 2A,B). Therefore, almost all of the tumor cells in the tissue from highly aggressive BL are positive for LAT1 (Fig. 2C). Immunoreactivity of LAT1 and Ki-67 antibodies were low in the indolent lymphoma cells such as FL and SLL (Fig. 2D,E). In the negative control (normal lymph node tissue), few cells were positive for LAT1 and Ki-67 (Fig. 2F). LAT1 was positive in both T cell-derived lymphoma and B cell-derived lymphoma, confirmed by double staining of CD20 or CD3 and LAT1 (Fig. 3A,B). The expression level of LAT1 has a good correlation efficiency with Ki67 expression in examined samples (r = 0.767, P < 0.001) (Fig. 4).

**Figure 5:**
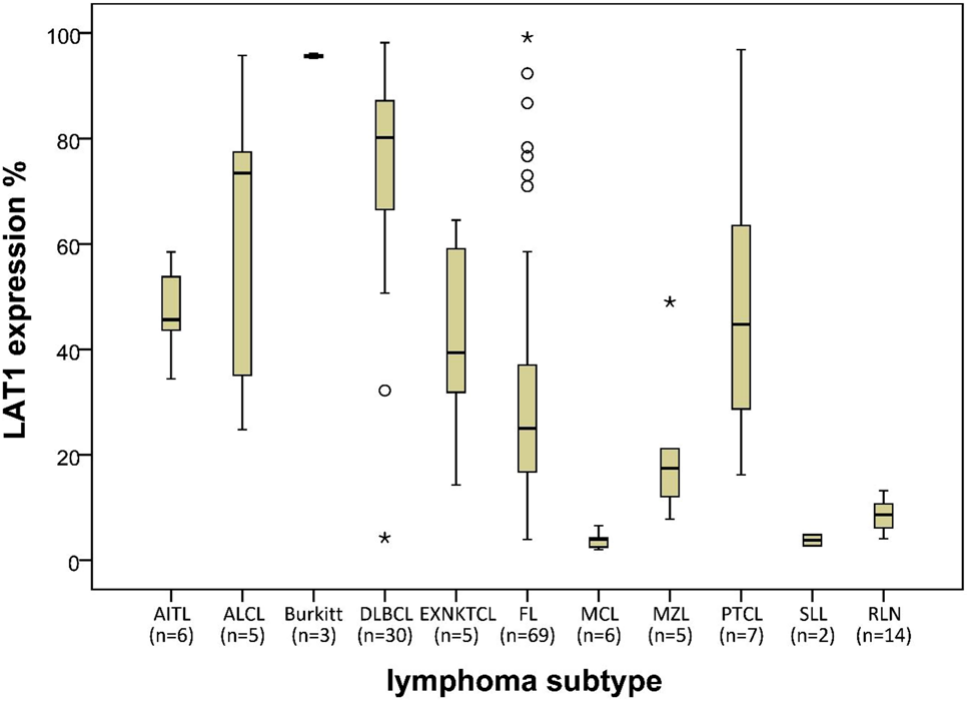
Comparison of LAT1 expression levels in 10 subtypes of NHL. AITL; Angioimmunoblastic T cell lymphoma, ALCL; Anaplastic large cell lymphoma, BL; Burkitt lymphoma, DLBCL; Diffuse large B cell lymphoma, ENKTCL; Extranodal Natural killer and T cell lymphoma, FL; Follicular lymphoma, MCL; Mantle cell lymphoma, MZL; Marginal zone lymphoma, PTCL; Primary T cell lymphoma, SLL; Small lymphocytic lymphoma, RLN: reactive lymph node.

The LAT1 expression level in NHL patients’ samples was ranged from 1.9 to 99.2%, with a median of 42.4% (SD ± 29.5%) (Table 2). For the most prevalent subtypes, DLBCL had a higher median LAT1 expression (80.1%) than that of FL (24.9%). The very indolent lymphoma, SLL had the lowest expression of LAT1, and the very aggressive lymphoma, BL had the highest expression (3.8 and 95.6%, respectively) (Fig. 5). At the reactive lymph nodes, the mean LAT1 expression of germinal center area and medulla or cortex area were 93.8% ± 5.02 and 8.4% ± 2.9, respectively.

**Table 2:** LAT1 expression in 138 patients with Non-Hodgkin’s lymphoma (WHO classification).

Lymphoma type	No. of patients	LAT1 expression level (%) Median (SD)	Range (%)
All types	138	42.4 (± 29.5)	1.9–99.2
**Diffuse large B-cell lymphoma (DLBCL)**	30 (21.7%)	80.1 (± 19.9)	4.2–98.2
GCB type	17	69.6 (± 23.1)	4.2–93.2
Non- GCB type	13	82.2 (± 11.6)	58.2–98.2
**Follicular lymphomas (FL)**	69 (50%)	24.9 (± 21.2)	3.9–99.2
Grade 1–2	41	22.6 (± 13.8)	3.9–76.6
Grade 2	6	26.9 (± 11.1)	10.8–40.6
Grade 3A	19	39.9 (± 23.8)	10.5–99.2
Grade 3B	3	81.2 (± 9.9)	73–92.3
Marginal zone B-cell lymphoma (MZL)	5 (3.6%)	17.4 (± 16.2)	7.7–49
Burkitt lymphoma (BL)	3 (2.2%)	95.6 (± 0.5)	95.2–96.2
Mantle cell lymphoma (MCL)	6 (4.3%)	3.9 (± 1.6)	1.9–6.5
Small lymphocytic lymphoma (SLL)	2 (1.4%)	3.8 (± 1.5)	2.7–4.8
Peripheral T-cell lymphoma (PTCL)	7 (5.1%)	44.7 (± 28.4)	16.2–96.8
Angioimmunoblastic T-cell lymphoma (AITL)	6 (4.3%)	45.6 (± 8.4)	34.4–58.4
Anaplastic large cell lymphoma (ALCL)	5 (3.6%)	73.4 (± 30.1)	24.8–95.7
Extranodal Natural Killer/T cell lymphoma nasal type (ENKTCL nasal)	5 (3.6%)	39.4 (± 24.5)	11.8–64.5

**Figure 6:**
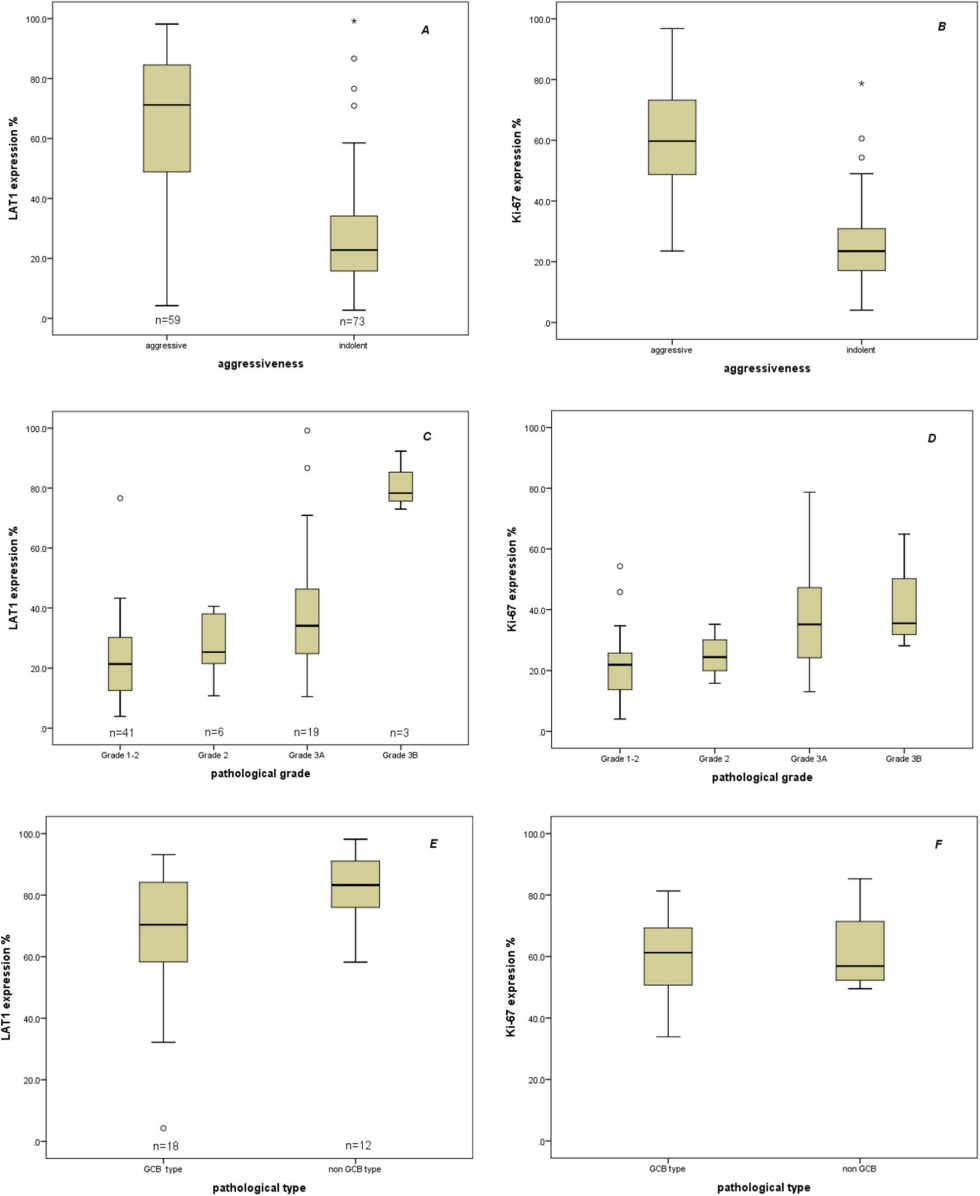
Correlation between expression levels of LAT1 and lymphoma grades. (**A**,**B**) LAT1 and Ki-67 expression level was significantly different between aggressive types and indolent types of NHL. (**C**,**D**) Pathological grades of FL were differed by an expression of LAT1 and Ki-67 levels between each other. (**E**,**F**) LAT1 and Ki-67 expression levels were not significantly different between GCB and non-GCB subtype of DLBCL.

Among the FL cases, median LAT1 expression level and pathological grade are increased in parallel, from 21.4% ± 13.8 for grade 1–2 to 25.4% ± 11.1 for grade 2, 34.1% ± 23.8 for grade 3A, and 78.3% ± 9.9 for grade 3B (Fig. 6C). Median LAT1 expression level of germinal center B-cell like (GCB) type of DLBCL and non-GCB type were 70.4% ± 22.4 and 83.3% ± 11.6, respectively (Table 2, Fig. 6E). In the cases of DLBCLs, 3 triple expressor lymphoma cases and 7 double expressor lymphoma cases were identified by the co-expression of cMYC, BCL2, and BCL6 (cutoff points for expression of cMYC protein (≥ 40%), BCL2 protein (≥ 50%), BCL6 protein (≥ 50%)). Median LAT1 expression level of triple expressor lymphoma, double expressor lymphoma, and other cases in which the co-expression of the proteins has not been determined were 92.6% ± 7.7, 84.2% ± 11.9 and 70.4 ± 21.8, respectively.

As the lymphoma grade, 73 cases were indolent and 59 cases were aggressive. In addition, 3 cases with FL grade 3B were classified as aggressive lymphoma. The median LAT1 expression of aggressive lymphoma (71.1% ± 25.1) was significantly higher than indolent lymphoma (22.7% ± 18.5) (Table 3; Fig. 6A). We also found that LAT1 expression and Ki-67 index are closely correlated to each other (Fig. 6A–F). ROC curve analysis established 43.5% as the threshold distinguishing indolent from aggressive lymphoma (area under the curve = 0.847, P < 0.001): 81.4% of aggressive lymphomas had a LAT1 expression level > 43.5%, 89% of indolent lymphomas had a LAT1 expression level < 43.5%. Thus, when this value was used, the LAT1 expression level could identify aggressive lymphomas with a sensitivity of 80.4% and a specificity of 85.5%.

**Table 3:** Median LAT1 expression level by lymphoma grade.

	No.of patients	LAT1 expression (%) Median (± SD)	Minimum (%)	Maximum (%)
Indolent lymphomas	73	22.7 (± 18.5)	2.7	99.2
Aggressive lymphomas	59	71.1 (± 25.1)	4.2	98.2
Mantle cell lymphomas	6	3.9 (± 1.6)	1.9	6.5
Total	138	42.4 (± 29.5)	1.9	99.2

### Correlation between LAT1 expression and clinical and laboratory parameters

Analysis with Spearman’s rank correlation showed that the LAT1 expression level was significantly correlated with Ki-67 index (r = 0.767, P < 0.001) and Lymphoma grade (r = 0.656, P < 0.001). In addition, LAT1 expression was weakly associated with serum level of LDH (r = 0.283, P = 0.001) and the status of patients (r = − 0.250, P < 0.001). There was no significant association between LAT1 expression level and other variables (patient gender or age, cell type, cancer stage, IPI or FLIPI2 score, extranodal and bone marrow involvement, cytogenetic abnormality, and hemoglobin level) (Table 4). In the indolent lymphomas (n = 73), LAT1 expression level was positively related to the Ki-67 index (r = 0.640, P < 0.001) and serum LDH level (r = 0.382, P = 0.001), and negatively associated with patient survival span (r = − 0.426, P < 0.001). In the aggressive lymphomas (n = 59), the LAT1 expression level was significantly correlated with Ki-67 index (r = 0.498, P < 0.001). We also observed a similar tendency in the correlation between the LAT1 expression level and OS in 59 patients with aggressive NHL, it was not statistically significant (spearman correlation coefficient was 0.134, P = 0.13). We are planning to collect a larger sample size for them in a future study.

**Table 4:** Correlation between the LAT1 and other variables by Spearman’s rank test.

Variables	Spearman γ	P-value
Ki-67 index	0.767	< 0.001*
Lymphoma grade	0.656	< 0.001*
Survival	− 0.324	< 0.001*
Serum LDH	0.283	0.001*
Cell type (B or T and NK cell)	0.155	0.069
Gender	− 0.125	0.146
Age	0.103	0.231
Cancer stage	− 0.034	0.691
HGB level	0.116	0.177
IPI score (among the cases another than FL n = 69)	0.071	0.563
FLIPI2 score (among FL cases n = 69)	− 0.037	0.761
BMI	− 0.019	0.825
Extranodal involvement	− 0.081	0.344
Cytogenetic abnormality	− 0.106	0.240

BMI, bone marrow involvement; HGB, hemoglobin; Serum LDH, serum lactate dehydrogenase.

*P-value < 0.05 indicates a significant difference.

Among the FLs, the LAT1 expression level was correlated with pathological grade (r = 0.473, P < 0.001). In the cases of DLBCLs, the LAT1 expression level was related with triple and double expressor type (r = 0.397, P < 0.05) and cMYC expression (r = 0.378, P < 0.05). Whereas, pathological subtypes GCB and non-GCB subtypes had no relationship with LAT1 expression level (r = 0.338, P = 0.068).

### Correlation between LAT1 expression and survival parameters

The mean duration of follow-up was 52.5 ± 36.3 months (range, 2–179 months). The nine patients with FL in limited stage (CS I or II) who did not need immediate treatment were on medical observation only. All of the other patients (n = 129) were received conventional chemotherapy, and 18 patients underwent autologous or allogeneic hematopoietic stem cell transplantation after the conventional chemotherapy. A combination of chemo- and radiation therapy was administrated to 23 patients.

**Figure 7:**
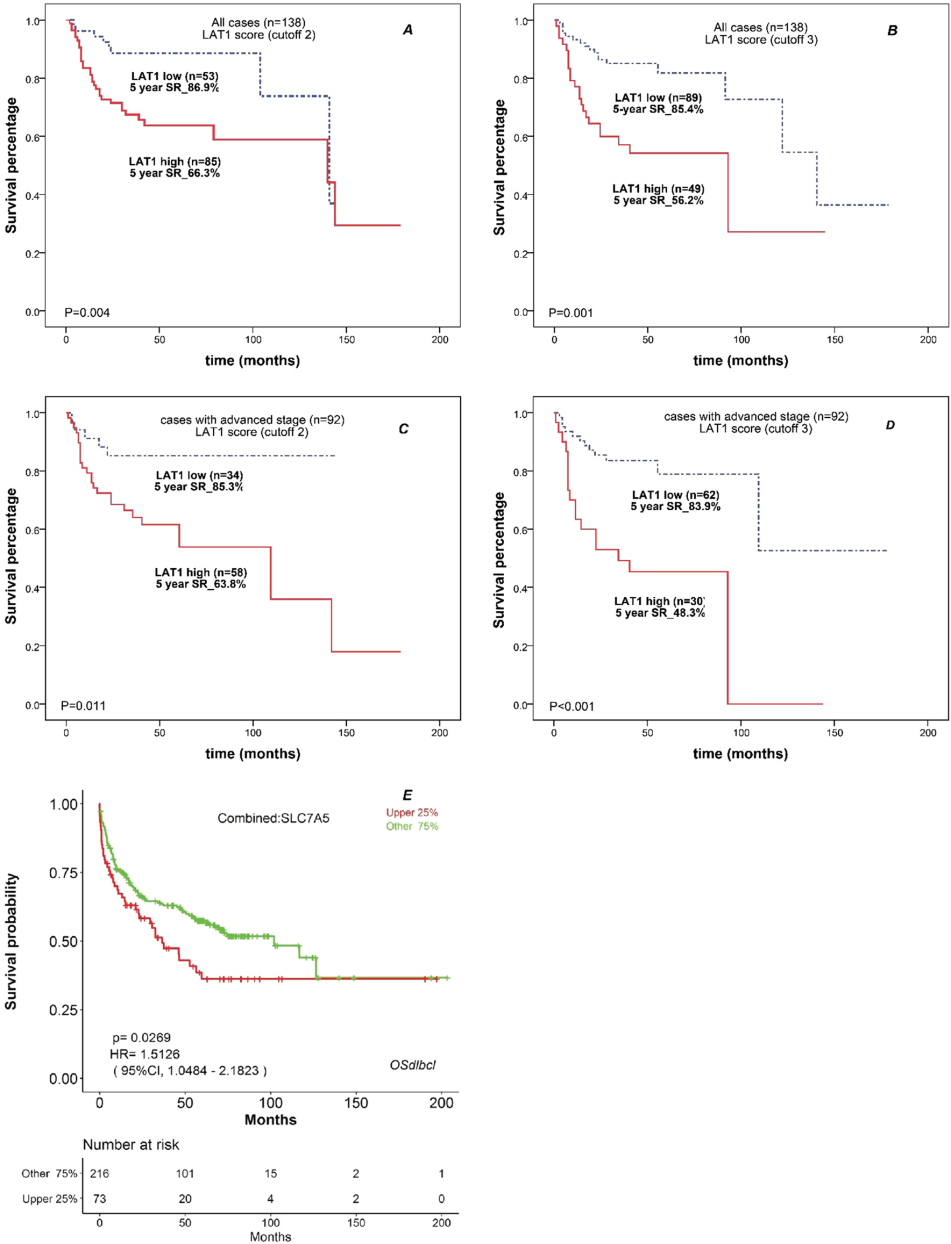
Higher LAT1 expression is a diagnostic marker for the poorer survival rate of patients with NHL. (**A**,**B**) Overall survival curves of patients with NHL divided by LAT1 scores with cutoffs of 2 (**A**) and 3 (**B**). (**C**,**D**) Overall survival curves of patients with advanced stage of NHL divided by LAT1 scores with cutoffs of 2 (**C**) and 3 (**D**). In all log-rank tests, outcomes were significantly poorer in the LAT1-high group than in the LAT1-low group. (**E**) Kaplan–Meier survival curve according to the gene expression of SLC7A5 in OSdlbcl web server. The survival analysis used the combined cohort and gene expression cutoff value set of 25%. The OS of the group with cutoff value upper 25% was poorer than the other group.

LAT1 expression level was reversely correlated with the entire NHL patients’ survival by analysis with Spearman’s rank test (r = − 0.324, P < 0.001). To dissect more in detail, all cases were classified into 2 groups with the LAT1 cut off score 2 (score 2: no more than 25% of cells were positive with LAT1); LAT1-low group (score 1 and 2; 53 patients) and LAT1-high group (score 3 and 4; 85 patients), and their survival were analyzed. The Kaplan–Meier estimator shows a significant difference in survival (P = 0.004, Fig. 7A). The 5-year survival rate of OS for LAT1-low and LAT1-high groups were 86.9% and 66.3%, respectively. When we focused on the group with the highest LAT1 score (score 3: more than 50% of cells were positive with LAT1), the 5-year survival rate of OS for LAT1-highest group was 56.2% and significantly lower than that of groups with LAT1 score 1–3 (85.4%, P < 0.001, Fig. 7B). As the cancer stage, total 92 patients were initially diagnosed at the advanced stage (CS III or IV) of lymphoma. Among these patients with advanced stage, when categorized with the LAT1 cutoff score as score 2 and 3, the Kaplan Meier plots in Fig. 7C,D showed that OS in the LAT1 high group was shorter than that in the LAT1 low group (P = 0.011 and P < 0.001, respectively).

Next, we examined whether LAT1 expression affects the OS in each subtype of NHL. In our study, the most prevalent subtype of NHL was FL (n = 69), followed by DLBCL (n = 30). Other subtypes were less than 10/group and the numbers were too small for the analysis, so we performed the OS analysis with FL and DLBCL. First, we divided the FL into two groups, Q4 which contains the highest quartile of LAT1 expression (N = 17) and the rest Q1-Q3 which contains FL patients with lower expression of LAT1 (Supplementary Figure 1A). The LAT1 Q4 tended to have a shorter OS, but it was not statistically significant (*p* = 0.31). We next compared the OS of the FL patients with Q4 (highest quartile: n = 17) or Q1 (lowest quartile: n = 17) of LAT1 expression (Supplementary Figure 1B). In this case, the tendency was more obvious, and the FL patients with higher LAT1 expression showed shorter OS. However, there was not a statistically significant difference (*p* = 0.14). Next, we divided the DLBCL patients into two groups by the LAT 1 expression level. When we compare the LAT1 highest Q4 group (n = 8) and LAT1 low group (Q1-Q3: n = 22), the LAT1 high group showed a tendency to have shorter OS, but it was also not statistically significant (*p* = 0.33) (Supplementary Figure 1C). Because we observed the same tendency that patients with higher LAT1 expression have shorter OS in both FL and DLBCL, we speculated that LAT1 expression level is a negative prognostic factor for NHL. However, we could not get statistically significant differences presumably due to the small number of patients. Therefore, we next investigated the effect of LAT1 expression in a publicly available database for NHL. Online Survival analysis web server Diffuse Large B-Cell Lymphoma (OSdlbcl) is based on cohorts of The Cancer Genome Atlas (TCGA) and the Gene Expression Omnibus (GEO) databases, to assess the prognostic value of individual genes^19^. According OSdlbcl, LAT1/SLC7A5 gene expression percentage was significantly related to overall survival of patients with DLBCL. OS of the group with SLC7A5 gene expression percentage higher than 25% was lower than the other group’s OS (P > 0.05, Fig. 7E). Univariable analysis revealed that unfavorable variables predicting OS were age > 60, decreasing hemoglobin level, high serum LDH level and high LAT1 expression (Table 5). Multivariable analysis showed that the patient's age and LAT1 expression were independently prognostic of OS.

**Table 5:** Cox hazard analysis of overall survival in NHLs (n = 138).

Variables	Cut off	Univariate analysis			Multivariate analysis		
		HR	95% CI	P value	HR	95% CI	P value
Age	60	4.4	1.82–10.49	0.001*	3.5	1.42–8.46	0.006*
Gender	Male/female	0.6	0.3–1.09	0.08			
CS	Limited/advanced	1.4	0.68–2.75	0.371			
HGB level	Normal/decreased	2.8	1.46–5.33	0.002*	1.9	0.99–3.37	0.05*
Serum LDH	Normal/increased	2.5	1.32–4.91	0.005*	1.6	0.84–3.22	0.15
BMI	No/yes	1.4	0.98–2.03	0.06			
Extranodal involvement	No/yes	1.2	0.63–2.22	0.613			
Cytogenetic abnormality	No/yes	0.9	0.58–1.39	0.629			
Ki-67 expression	70% low/high	0.7	0.25–2.06	0.605			
LAT1 expression	Low/high	2.9	1.35–6.41	0.006*	2.4	1.10–5.23	0.028*

BMI, bone marrow involvement; CI, confidence interval; CS, cancer stage; HGB level, hemoglobin level; HR, hazard ratio; serum LDH, serum lactate dehydrogenase.

*P-value < 0.05 indicates a significant difference.

### LAT1 as a therapeutic target of NHL

**Figure 8:**
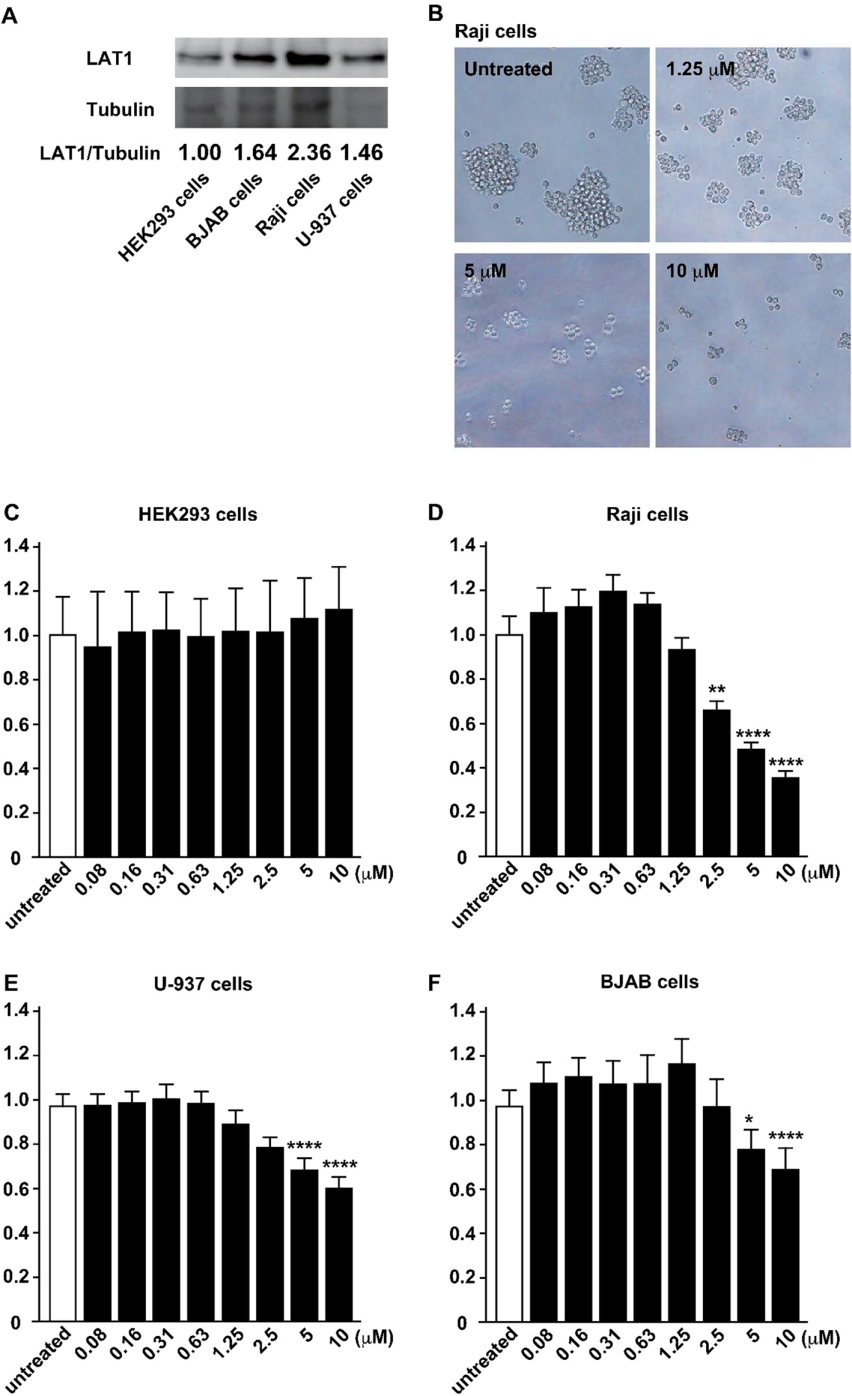
The effect of LAT1 inhibitor JPH203 on the viability of lymphoma cell lines and HEK293 cells. (**A**) Western blot analysis of LAT1 and tubulin. The LAT1/tubulin ratio was normalized to HEK293 cells. (**B**) Colony formation of Raji cells treated with the indicated concentration of JPH203. (**C**–**F**) The viability of Raji cells, BJAB cells, U-937 cells and HEK293 cells were analyzed after 8 different dozes of JPH203 treatment for 72 h and determined by Cell-titer glo 2. Significant cell death was observed at the concentration of 2.5 µM, 5 µM and 10 µM of JPH203 in Raji cell line. *P < 0.05; **P < 0.01; ****P < 0.0001.

Because we detected LAT1 expression in all NHL samples examined and found a positive correlation between LAT1 expression and lymphoma grade, we hypothesized that inhibition of LAT1 might suppress the growth of lymphoma cells, and inhibitors of LAT1 can be novel therapeutics for NHL. Inhibitors of LAT1 are extensively developed for the treatment of malignant tumors and JPH203, the most potent small compound inhibitor of LAT1, is in clinical trials for solid tumors such as colorectal cancer and biliary tract cancer^20^. To investigate whether inhibition of LAT1 can suppress lymphoma cell proliferation, we examined three human lymphoma cell lines, Raji cells, BJAB cells, and U-937 cells. HEK293 cells were used as a negative control cell line^14^. First, we performed western blot analysis of LAT1 and confirmed that these lymphoma cell lines expressed a high level of LAT1, and especially Raji cells showed the highest expression (Fig. 8A). Next, we treated these cell lines with different concentrations of JPH203. When we escalated the concentration, it significantly inhibited colony formation of Raji cells (Fig. 8B). JPH203 also inhibited proliferation of all three lymphoma cell lines but not that of HEK293 cells (Fig. 8C–F), suggesting that inhibition of LAT1 is a potent therapeutic modality for NHL.

## Discussion

NHL is the most prevalent hematologic malignant disease, affecting more than a half-million people every year. Recent advances in the development of molecular targeted therapies such as rituximab have drastically improved the outcome and quality of life of patients, however, the overall survival rate in some aggressive types of NHL is still pretty low and further investigation for finding novel prognostic markers as well as therapeutic targets are aspired. Because NHL consists of highly heterogeneous groups of diseases, precise molecular characterization is necessary for determining optimal chemotherapeutic regimens. In this study, we revealed that LAT1 expression correlated with disease lymphoma grade and the LAT1 expression level inversely correlated with patient survival, suggesting that LAT1 can be used as a prognostic marker for NHL. Furthermore, we demonstrated that inhibition of LAT1 by a specific inhibitor JPH203 prevents lymphoma cell proliferation, thus LAT1 can be a possible therapeutic target for NHL.

NHL is a highly heterogeneous disease containing more than 100 groups of hematologic malignancies. The molecular diagnosis of NHL is proceeded by a combination of several biological markers including cell surface markers, such as CD3 for T cells, CD20 for B cells, CD56 for NK cells, and Ki-67 index which indicates the proliferation of tumor cells. The expression of Ki-67 antigen is confirmed in cells at active phases of the cell cycle (G_1_, S, G_2_ and mitosis) whereas its expression is absent in the static G_0_ phase. The Ki-67 expression rate correlates with NHL grade and thus is useful for a prognostic predictor of NHL^4,5^. When tumor cells proliferate, they require a large amount of nutrition to produce necessary proteins. To uptake amino acids across the plasma membrane, tumor cells express LAT1 which transports large neutral amino acids. The restricted expression profile of LAT1 to malignant tissues makes it a favorable marker for the diagnosis of NHL. Because the LAT1 expression is high in actively proliferating tumor cells, its expression signature was expected to be similar to that of Ki-67, and indeed LAT1 expression has a good correlation efficiency with Ki-67 expression (Fig. 4). Among various types of NHL, FL and DLBCL are the most common NHL^3^ and in our study, 71.7% of total cases were diagnosed as FL and DLBCL. Clinically, the majority of patients with DLBCL have a poor prognosis compared with patients with FL due to their aggressive tumor growth, and the mean LAT1 expression level was also high in DLBCL (80.1%) than in FL (24.9%). BL is notorious for its aggressiveness and in our study, although the patient number was small (n = 3), the LAT1 expression was high in all three cases (> 90%), suggesting that LAT1 expression level reflects disease aggressiveness. The co-expression of LAT1 and CD3 we well as CD20 indicated that high LAT1 expression could be observed despite the origin of tumor cells.

In the 10 type of NHL samples consisting of 138 patients we examined, we found that the LAT1 expression rate was widely varied in each case (1.9–99.2%). We did not find any LAT1 negative case, probably because some of the normal lymphocytes at the activated state also express LAT1. Aggressive lymphoma subtypes, DLBCL, BL, and ALCL, have higher expression rates of LAT1 (80.1%, 95.6% and 73.4%, respectively) than that of indolent lymphoma types, FL and SLL (24.9% and 3.8%, respectively). In the current study, we noted a statistically significant increase in the median LAT1 with an increase in lymphoma grade (P < 0.001). On ROC curve analysis, an index value of 43.5% differentiated indolent from aggressive lymphoma (AUC = 0.847, P < 0.001) with a sensitivity of 80.4% and a specificity of 85.5%. This confirmed that LAT1 is a potential marker for determining the cellular proliferation rate. This idea is also supported by the fact that the LAT1 expression level was significantly correlated to the expression level of the Ki-67 (r = 0.767, P < 0.001) which indicates a cellular proliferation rate. We found that activated lymphocytes in the germinal center of reactive lymph nodes were strongly positive for LAT1. Therefore, LAT1 immunohistochemistry staining should be used after the initial distinction of lymphoma as histopathology.

Especially in Follicular lymphomas (n = 69), the median LAT1 expression level and pathological grade are increased in parallel, from 21.4% ± 13.8 for grade 1–2 to 78.3% ± 9.9 for grade 3B. The LAT1 expression level was also significantly correlated with patient survival (r = − 0.400, P = 0.001). Thus, LAT1 could be used as a new marker to distinguish pathological grade and to predict prognosis in FL cases. In DLBCLs (n = 30), 29 samples were classified into the LAT1 high group, however, there was no association between LAT1 and pathological subtype and survival. By the OS analyses with our limited number of NHL patients, we could not find a statistically significant difference of OS between the LAT1 high group and LAT1 low group in each subtype, however, the correlation between LAT1 gene expression level and OS of patients with DLBCL was confirmed by the online database, OSdlbcl, substantiating our hypothesis and further study with a large number of NHL patients for each subtype is warranted. Another important finding of our study was that triple and double expresser lymphoma types and the expression of cMYC protein were significantly associated with LAT1 expression level in cases with DLBCL. Several previous studies^21–25^ revealed that high expression of LAT1 is associated with malignant tumor aggressiveness and poor prognosis. Likewise, our results indicated that overexpression of LAT1 was an independent prognostic factor of poor survival by multivariable analysis.

JPH203 is a potent and selective small inhibitor for LAT1 and its robust anti-tumor activity is now examined in a clinical trial for colorectal cancer, biliary tract cancer, and pancreatic cancer^20^. Because NHL tissues examined have high expression of LAT1 and JPH203 treatment efficiently killed lymphoma cells in vitro, it appears that JPH203 might be a potent therapeutic against NHL. Because conventional chemotherapeutics for NHL target proliferating tumor cells non-specifically by alkylation of DNA (such as doxorubicin) or destabilization of microtubules (such as vincristine), they cause severe side effects in actively proliferating normal tissues including bone marrow suppression and mucosal epithelial damages. The expression of LAT1 in normal tissues is relatively limited and also they express other LAT species which are functionally redundant to LAT1, they can escape the toxicity from LAT1 inhibition. On the other hand, the growth of tumor cells is dependent on LAT1, and thus JPH203 is expected to suppress tumor proliferation without these harmful events. Immunohistochemical analysis showed that LAT1 expression correlates with lymphoma grade, so inhibition of LAT1 is expected to be effective for aggressive types of NHL with higher cell proliferation rate.

In conclusion, we found that LAT1 expression is an excellent prognostic marker for NHL and its expression reflects lymphoma grade as well as tumor proliferation. The tumor-specific expression of LAT1 makes it a favorable chemotherapeutic target for NHL and inhibition of LAT1 will be a potential therapeutic modality for NHL.

## Supplementary Information

Supplementary Information.

## Data Availability

All data generated or analyzed during this study were included in the article.
