# Supplementary material for: Expression of L-type amino acid transporter 1 is a poor prognostic factor for Non-Hodgkin’s lymphoma
Source: Sci Rep. 2021 Nov 4;11:21638. doi: 10.1038/s41598-021-00811-8 (PMC8569019; doi:10.1038/s41598-021-00811-8)

Supplementary information

Expression of L-type amino acid transporter 1 is a poor prognostic factor for Non-Hodgkin's lymphoma.

Authors: Narangerel Jigjidkhorloo[1,2], Kohsuke Kanekura*[1], Jun Matsubayashi[3], Daigo Akahane[4], Koji Fujita[1], Keiki Oikawa[1], Atsushi Kurata[1], Masakatsu Takanashi[1], Hitoshi Endou[5], Toshitaka Nagao[3], Akihiko Gotoh[4], Oyundelger Norov[2], Masahiko Kuroda*[1]

Affiliations:

[1] Department of Molecular Pathology, Tokyo Medical University, 6-1-1 Shinjuku, Shinjuku-ku, Tokyo, 160-8402, Japan.

[2] Center of Hematology and Blood & Marrow Transplantation, The First Central Hospital of Mongolia, Ulaanbaatar, 14210, Mongolia.

[3] Department of Anatomical Pathology, Tokyo Medical University Hospital, 6-7-1 Nishi-Shinjuku, Shinjuku-ku, Tokyo, 160-0023, Japan.

[4] Department of Hematology, Tokyo Medical University Hospital, 6-7-1 Nishi-Shinjuku, Shinjuku-ku, Tokyo, 160-0023, Japan.

[5] J-Pharma Co., Ltd., 75-1 Ono-cho, Tsurumi-ku, Yokohama, Kanagawa 230-0046, Japan.

*Correspondence to: Kohsuke Kanekura, M.D., Ph.D. or Masahiko Kuroda, M.D., Ph.D.

Kohsuke Kanekura, M.D., Ph.D, Assistant Professor, Department of Molecular Pathology, Tokyo Medical University, 6-1-1, Shinjuku, Shinjuku-ku, Tokyo, 160-8402, Japan.
Tel: +81-3-3351-6141
E mail: kanekura@tokyo-med.ac.jp

Masahiko Kuroda, M.D., Ph.D, Professor, Department of Molecular Pathology, Tokyo Medical University, 6-1-1, Shinjuku, Shinjuku-ku, Tokyo, 160-8402, Japan.
Tel: +81-3-3351-6141
E mail: kuroda@tokyo-med.ac.jp

Supplementary Figure 1. Correlation between expression levels of LAT1 and OS of FL or DLBCL. (A) The patients with FL (N=69) were divided into quartiles by the expression level of LAT1. The OS of the highest group Q4 (N=17) were compared with the OS of Q1-Q3 (N=52). (B)The OS of FL patients with Q4 (highest quartile: n=17) was compared with the OS of FL patients with Q1 (lowest quartile: n=17). (C) The patients with DLBCL (N=30) were divided into quartiles by the expression level of LAT1. The OS of the highest group Q4 (N=8) were compared with the OS of Q1-Q3 (N=22).

Supplementary Figure 1

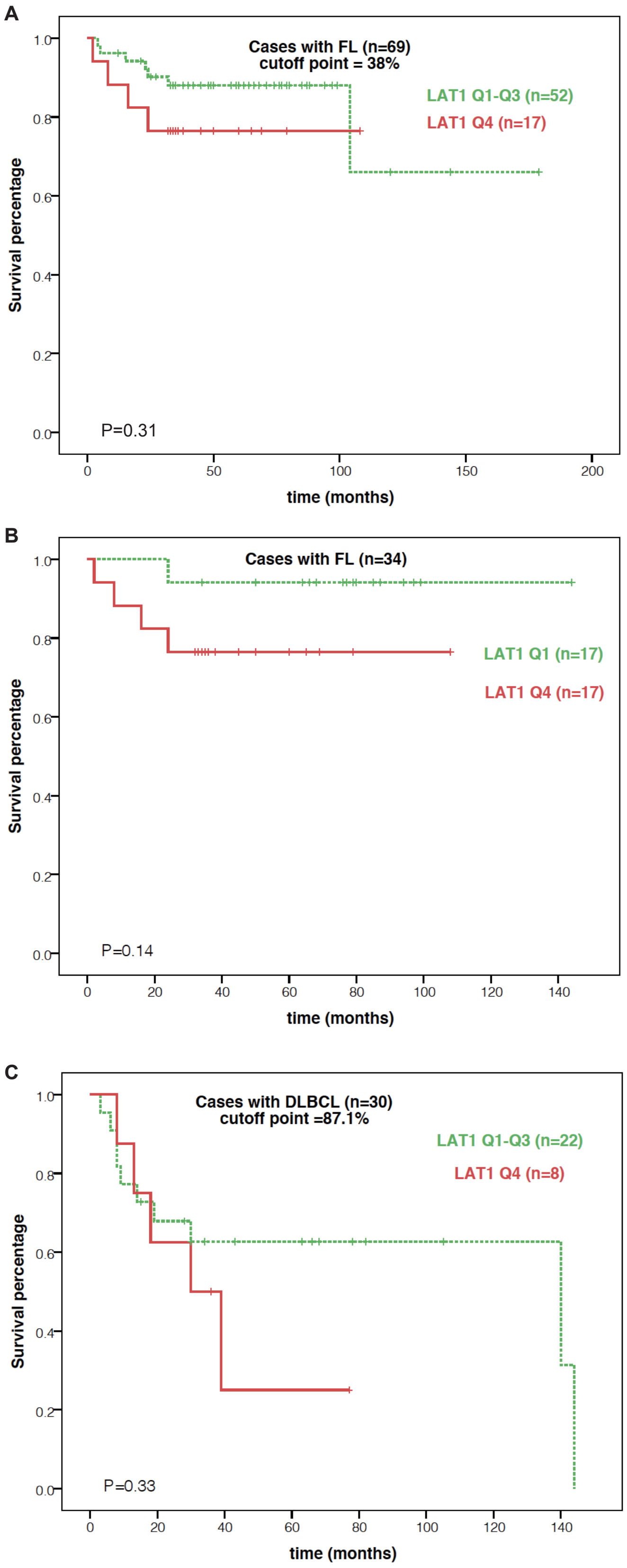

Supplement: Supplementary file 1 — Supplementary Information. [file 41598_2021_811_MOESM1_ESM.pdf]
